# Emicizumab prophylaxis in current haemophilia A care in the Czech Republic—data from the Czech National Haemophilia Programme Registry

**DOI:** 10.1016/j.rpth.2025.103215

**Published:** 2025-10-10

**Authors:** Ester Zápotocká, Petra Ovesná, Bohumír Blažek, Zuzana Čermáková, Zdeňka Hajšmanová, Radomíra Hrdličková, Petr Birke, Daniela Procházková, Petr Smejkal, Jan Blatný, Czech National Haemophilia Programme

**Affiliations:** 1 Faculty of Medicine, Masaryk University, Brno, Czech Republic; 2 Department of Paediatric Haematology and Oncology, University Hospital Motol and Second Faculty of Medicine, Charles University, Prague, Czech Republic; 3 Institute of Biostatistics and Analyses, Ltd., Brno, Czech Republic; 4 Department of Paediatrics, University Hospital Ostrava, Czech Republic; 5 Blood Centre University Hospital Ostrava, Ostrava, Czech Republic; 6 Department of Haematology, Institute of Clinical Biochemistry and Haematology, Charles University, Pilsen, Czech Republic; 7 Department of Pediatrics, Palacky University and University Hospital, Olomouc, Czech Republic; 8 Department of Paediatrics, J.E. Purkyne University, Usti nad Labem, Czech Republic; 9 Department of Clinical Haematology University Hospital Brno, Brno, Department of Laboratory Methods, Masaryk University, Brno, Czech Republic; 10 Department of Paediatric Haematology and Biochemistry, University Hospital Brno, Brno, Czech Republic

**Keywords:** annual bleeding rate, consumption, emicizumab, haemophilia A, prophylaxis, zero bleeds

## Abstract

**Background:**

Nonfactor therapy with emicizumab has become an important part of the haemophilia A treatment landscape recently.

**Objectives:**

We aimed to analyze data on the transition from previous treatment regimens to emicizumab in routine clinical practice in the Czech Republic.

**Methods:**

Retrospective analysis of data from the Czech National Haemophilia Programme (CNHP) registry of all consecutive pediatric and adult persons treated with emicizumab prophylaxis. We evaluated bleeding control, injection frequency, and drug consumption in the period before and after emicizumab initiation.

**Results:**

Seventy-three persons with severe haemophilia A, median (IQR) age 4 (1-18) years, treated with emicizumab were reported in the CNHP registry up to the end of 2023. Two age categories were used for further stratification, 0-11 (*n* = 45) and 12+ (*n* = 28) years. For the whole group, we observed a significant reduction in total annualized bleeding rate (ABR) from a mean of 3.44 to 0.46 (*P* < .001), in joint ABR from 0.70 to 0.27 (*P* = .004), and in spontaneous ABR from 1.18 to 0.19 (*P* = .002) after starting emicizumab. The proportion of persons with no reported bleeding increased from 26.0% to 60.3% (*P* < .001). A sub-analysis, focused on inhibitors presence (present in 27 cases) and age, showed a more significant effect of emicizumab switch in observed parameters in persons with inhibitors and in the younger age group.

**Conclusion:**

The retrospective analysis of nation-wide data from the CNHP registry supports the fact that emicizumab has the potential to reduce bleeding and to provide an opportunity for a significant proportion of patients, with and without factor VIII inhibitors, to achieve zero bleeds.

## Essentials

• Emicizumab has become an important part of the treatment landscape for haemophilia A.
• Analysis of data from the Czech national registry of all consecutive persons on emicizumab.
• We observed significant increase in the proportion of persons with zero bleeds on emicizumab.
• Our data strongly support preference of emicizumab prophylaxis in various clinical scenarios.

## 1 Introduction

Haemophilia A (HA), an inherited bleeding disorder caused by factor (F)VIII deficiency, is known to be a debilitating disease if untreated. Especially in developed countries, prophylaxis represents the gold standard of care for the majority of persons with severe haemophilia and those with the less severe disease form who are more likely to bleed [1]. An efficiently adjusted prophylactic treatment regimen significantly reduces bleeding episodes and has great potential to protect joints from otherwise inevitable impairment caused by recurrent macro or micro bleeds into the articular cavity [2].

The treatment landscape for haemophilia has evolved significantly over the last 10 years [3], mainly due to the advent of new treatment options. In addition to prolonging the biological half-life of the FVIII molecule using different technologies (e.g. PEGylation or Fc fragment IgG binding), which has led to the development of a new group of drugs called extended half-life factors (EHLs) [4], research has also focused on the use of other approaches to reduce the risk of bleeding in haemophilia persons (originally in persons with inhibitors), referred to as nonfactor therapy [5]. Within this group of new treatment alternatives, so-called rebalancing drugs are in various stages of clinical research, while the antibody mimicking FVIII, emicizumab, has already entered clinical practice [5].

The first pivotal studies with emicizumab in persons with inhibitors showed a significant reduction in treated bleeding episodes and an improved quality of life in these complicated cases, compared both to persons previously treated on demand and to previous prophylaxis with bypassing agents (BPA) [[6], [7], [8]]. Very similar benefits in terms of treated bleed reduction have also been shown in adult and younger persons without inhibitors [9,10]. In addition to the observed favorable efficacy and safety profile of emicizumab, its potential to reduce significantly the treatment burden due to subcutaneous administration and prolonged dosing frequency is highly appreciated.

Emicizumab has been available in the Czech Republic since the end of 2018. In our study, we aimed to describe Czech real clinical practice/experience after the introduction of emicizumab, and current trends in haemophilia A treatment.

## 2 Materials and Methods

Data from the Czech National Haemophilia Programme (CNHP) registry were analyzed. The registry contains data on persons with haemophilia in all but one of the Czech CCC and HTC centres for adults. All pediatric centers are involved in the registry. Data are collected annually via a web-based system.

All persons treated with emicizumab for at least 6 months, with a data cut-off date of 31 December 2023, were included in the analysis. Treatment outcomes during emicizumab prophylaxis were compared with a previous treatment period (pre-emicizumab). This period was defined as a consistent period on a single type of treatment (either prophylaxis or immune tolerance therapy) prior to the initiation of emicizumab. For on-demand regimens, the before period started 24 months prior to the start of emicizumab, or at the date of birth if emicizumab was started before the age of 2 years. Therefore, the pre-emicizumab period varied in length between cases (similarly to the emicizumab period).

The following parameters were evaluated: age, sex (male/female), treatment before and after initiation of emicizumab (factor VIII and/or BPA dose, dosing interval, weekly FVIII and/or BPA consumption on prophylaxis, consumption and dosing of emicizumab), and bleeding management (number/type of bleeds and FVIII and/or BPA consumption).

To assess bleeding control on the respective therapy, we calculated and evaluated the overall annualized bleeding rate (ABR) and different subtypes of bleeding: spontaneous (AsBR), traumatic (AtBR), and joint (AjBR).

We also evaluated the safety of emicizumab treatment with regard to the development of antidrug antibodies against emicizumab (either by repeated aPTT screening in most of the centers or by direct antibodies assessment in one center) , the occurrence of other reported adverse events and the termination and eventual reason of emicizumab prophylaxis.

Consumption (of respected medications) was counted per kg of weight for comparability. Bleeding frequency was annualized (ABR) due to the different length of follow-up in the assessed persons. Standard descriptive statistics were used to describe the data—absolute and relative frequencies for categorical variables and mean, SD, median, and interquartile range (IQR 0.25-0.75 percentiles) for continuous variables. Paired comparisons (pre-emicizumab vs. emicizumab) were performed using the McNemar or Wilcoxon paired tests.

## 3 Results

### 3.1 Demographics

The total number of haemophilia A persons followed in the CNHP registry by the end of 2023 was 641, of whom 247 were children (100 with severe HA and 147 with nonsevere HA) and 394 were adults (147 with severe HA and 247 with nonsevere HA).

A total of 79 persons (all male) treated with emicizumab, were reported in the CNHP registry by the end of 2023, of whom 73 were teated for at least 6 months. The median age at the beginning of emicizumab treatment was 4 (IQR, 1-18) years. Three age categories (0-4; 5-11; and 12+) were originally considered (Table 1), but only 5 patients started emicizumab treatment at the age of 5-11 years; therefore, 2 categories, 0-11 and 12+ years, were finally used for further stratification. 61.6% of all cases were children less than 12 years of age, with a predominance of young children (0-4 years) in both the noninhibitor (70.4%) and inhibitor (56.5%) groups. At the time of switch to emicizumab, 27 (36.9%) persons had active FVIII inhibitors: 10 were on prophylaxis with BPA, 9 were treated on demand, and 8 were treated/started on immune tolerance therapy. Out of 46 (63.1%) persons without inhibitors, 32 were on factor prophylaxis (Table 2) and 14 were treated on demand (before start of the regular primary prophylaxis—either before the age of 2 years or before the second clinically significant joint bleed) [11], including 4, who were previously untreated patients (PUPs) and 9 with previously less than 5 exposure days (ED) to FVIII concentrates—so call minimally treated patients (MTPs). In 2 infants emicizumab was started following intracranial haemorrhage after initial treatment and stabilization with factor therapies, aiming for a stable level of prophylaxis without low trough levels. No inhibitors developed in these children.

**Table 1:** Patients’ characteristics.

	Overall	Age at emicizumab start (y)		Inhibitor at emicizumab start	
	*N* = 73	0-11 *n* = 45	12+ *n* = 28	Yes *n* = 27	No *n* = 46
Age at emicizumab start					
Mean ± SD	14 ± 19	2 ± 2	33 ± 19	14 ± 22	14 ± 17
Median (IQR)	4 (1-18)	1 (1-3)	33 (16-42)	3 (1-15)	5 (1-20)
Age at emicizumab start					
0-4	40 (54.8%)	40 (88.9%)	0 (0.0%)	17 (63.0%)	23 (50.0%)
5-11	5 (6.8%)	5 (11.1%)	0 (0.0%)	2 (7.4%)	3 (6.5%)
12+	28 (38.4%)	0 (0.0%)	28 (100.0%)	8 (29.6%)	20 (43.5%)
Weight (kg)					
Mean ± SD	39 ± 34	15 ± 6	79 ± 20	37 ± 36	41 ± 33
Median (IQR)	19 (12-71)	13 (11-17)	83 (60-93)	19 (13-57)	23 (11-74)
Severity of HA					
Severe phenotype	73 (100.0%)	45 (100.0%)	28 (100.0%)	27 (100.0%)	46 (100.0%)
Inhibitor at emicizumab start	27 (37.0%)	19 (42.2%)	8 (28.6%)	–	–
Length of emicizumab prophylaxis (mo)					
Mean ± SD	30 ± 15	30 ± 15	30 ± 15	38 ± 16	26 ± 12
Median (IQR)	30 (18-43)	28 (18-46)	32 (20-39)	46 (22-51)	24 (18-34)
Min-Max	7-57	7-56	7-57	9-57	7-51
Treatment regimen before emicizumab					
PX	42 (57.5%)	18 (40.0%)	24 (85.7%)	10 (37.0%)	32 (69.6%)
PX + ITI	5 (6.8%)	4 (8.9%)	1 (3.6%)	5 (18.5%)	0 (0.0%)
ITI	3 (4.1%)	3 (6.7%)	0 (0.0%)	3 (11.1%)	0 (0.0%)
OD	23 (31.5%)	20 (44.4%)	3 (10.7%)	9 (33.3%)	14 (30.4%)
Length of follow-up before emicizumab (mo)					
Mean ± SD	19 ± 14	14 ± 10	27 ± 15	17 ± 11	21 ± 15
Median (IQR)	15 (9-24)	12 (8-19)	24 (17-39)	15 (7-24)	18 (9-29)
Min-Max	0.16-51	0.16-50	1.51-51	3.38-38	0.16-51

HA, haemophilia A; ITI, immune tolerance therapy; IQR, interquartile range; OD, on demand; PX, prophylaxis.

**Table 2:** Prophylaxis with FVIII regimens before switch to emicizumab prophylaxis—subgrouped by age and by FVIII class (SHL vs. EHL).

	Age group 0-11		Age group 12+	
	FVIII EHL*n* = 6	FVIII SHL*n* = 7	FVIII EHL *n* = 10	FVIII SHL *n* = 10
Frequency per week (d), mean ± SD; median (Q1-Q3)	1.89 ± 0.78; 2.00 (1.00-2.33)	1.69 ± 1.24; 1.00 (1.00-3.00)	2.01 ± 0.64; 2.00 (1.75-2.00)	2.11 ± 0.65; 2.00 (1.54-2.07)
Dose per kg (IU/week), mean ± SD; median (Q1-Q3)	75 ± 34; 68 (53-88)	38 ± 29; 21 (20-63)	63 ± 30; 57 (43-89)	62 ± 23; 55 (44-75)

EHL, extended half-life; FVIII, factor VIII; SHL, standard half-life.

The median follow-up in the pre-emicizumab period was 15 months; the median follow-up with emicizumab was 30 months, with a longer follow-up in persons with inhibitors (46 vs. 24 months), reflecting the fact that emicizumab was approved and reimbursed later in the Czech Republic for persons without inhibitors. Details are shown in Table 1.

### 3.2 Emicizumab dosing

The most frequently used emicizumab maintenance regimen was 3 mg/kg every 2 weeks (79.5%), followed by 1.5 mg/kg weekly (11.0%) and 6 mg/kg every 4 weeks (2.7%). Five persons were dosed out of the summary product characteristic recommendation with so-called whole vial approach [12,13]. There was no need for further dose adjustment of emicizumab (e.g. based on measured levels).

### 3.3 Bleeding

#### 3.3.1 Total cohort

The mean (median) number of the total ABR was 3.44 (1.37) prior to emicizumab initiation compared with 0.46 (0.0) with emicizumab prophylaxis. The reduction of ABR was statistically significant (*P* < .001). AjBR decreased from a mean of 0.70 to 0.27 (*P* = .004) and AsBR decreased from 1.18 to 0.19 per year (*P* = .002). The proportion of persons with no reported bleeding (zero bleeds) increased significantly on emicizumab (60.3% vs. 26.0% before emicizumab, *P* < .001), as was the case also for zero joint bleeds (82.2% vs. 64.4%, *P* = .045) and zero spontaneous bleeds (80.8% vs. 65.8%, *P* = .029). Details are shown in Table 3 and Figure. The high SD for ABR, especially in the pre-emicizumab period, suggests a skewed distribution of ABR, i.e. the existence of several cases (mostly with FVIII inhibitor presence) with extremely high bleeding rates who were also successfully treated with emicizumab.

**Table 3:** Annualized bleeding rates—total group.

	Before emicizumab	Emicizumab	*P*^a^
	*n*= 73	*n* = 73	
ABR			< .001
Mean ± SD	3.44 ± 9.29	0.46 ± 1.02	
Median (IQR)	1.37 (0.00-2.50)	0.00 (0.00-0.52)	
AjBR			.004
Mean ± SD	0.70 ± 1.58	0.27 ± 0.87	
Median (IQR)	0.00 (0.00-0.52)	0.00 (0.00-0.00)	
AsBR			.002
Mean ± SD	1.18 ± 3.31	0.19 ± 0.62	
Median (IQR)	0.00 (0.00-0.80)	0.00 (0.00-0.00)	
ABR			< .001
0	19 (26.0%)	44 (60.3%)	
<1	12 (16.4%)	20 (27.4%)	
1+	42 (57.5%)	9 (12.3%)	
AjBR			.045
0	47 (64.4%)	60 (82.2%)	
<1	13 (17.8%)	6 (8.2%)	
1+	13 (17.8%)	7 (9.6%)	
AsBR			.029
0	48 (65.8%)	59 (80.8%)	
<1	8 (11.0%)	10 (13.7%)	
1+	17 (23.3%)	4 (5.5%)	

ABR, annualized bleeding rate; AjBR, annualized joint bleeding rate; AsBR, annualized spontaneous bleeding rate; IQR, interquartile range.

a Wilcoxon signed rank test with continuity correction; McNemar's χ^2^ test.

**Figure:**
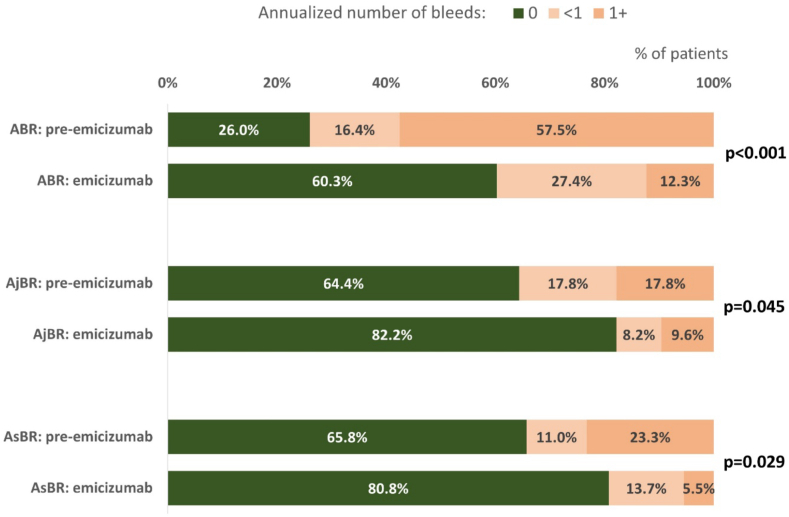
Annualized numbers of bleeds in total cohort. The graph compares the proportion of patients with zero, less than one and one and more than one bleed per year in both periods. ABR, annualized bleeding rate; AjBR, annualized joint bleeding rate; AsBR, annualized spontaneous bleeding rate.

#### 3.3.2 Age group 0-11, regardless of inhibitor status

The mean (median) total ABR in the pre-emicizumab period was 4.4 (1.5), while a statistically significant reduction was observed after the start of emicizumab with an ABR of 0.29 (0.0) (*P* < .001). The mean (median) AjBR and AsBR in the pre-emicizumab period was 0.38 (0.0) compared to 0.08 (0.0) with emicizumab (*P* = .055) and 1.33 (0.0) compared to 0.09 (0.0) with emicizumab (*P* = .018). There was also a statistically significant reduction observed in the achievement of zero bleeding (66.7% on emicizumab vs. 26.7% pre-emicizumab, *P* < .001).

#### 3.3.3 Age group 12+, regardless of inhibitor status

The mean (median) total ABR prior to emicizumab initiation was 1.90 (1.04) compared with 0.73 (0.11) after switching to emicizumab (*P* = .004). The proportion of persons with zero bleeds increased after switching to emicizumab (50.0% vs. 25.0%; *P* = .063). For the AjBR, the mean (median) value decreased from 1.21 (0.40) to 0.59 (0.0) with emicizumab (*P* = .024), AsBR dropped from 0.93 (0.0) to 0.35 (0.0); *P* = .052.

One adult person discontinued emicizumab and switched back to FVIII prophylaxis due to recurrent bleeds into a target joint that was already present prior to the initiation of emicizumab. This man was not obese but was involved in daily manual work. He underwent extensive testing, including tests for antidrug antibodies, which were negative. The reason for the change was therefore the insufficient clinical efficacy of the emicizumab treatment.

#### 3.3.4 Comparison by the presence of inhibitors

In the pre-emicizumab period, mean (median) total ABR was slightly higher in persons with inhibitors compared with persons without inhibitors (3.75 [1.51] vs. 3.27 [1.28]). Again, a significant reduction in total ABR was observed after emicizumab initiation, regardless of the presence of inhibitors, with mean (median) ABR 0.24 (0.0) in the inhibitor group (*P* < .001) and 0.6 (0.0) in the noninhibitor group (*P* < .001). The mean (median) AjBR and AsBR were significantly reduced in the inhibitor group with emicizumab (1.0 [0.0] before vs. 0.11 [0.0] with emicizumab [*P* = .018] and 1.74 [0.0] before vs. 0.04 [0.0] with emicizumab [*P* = .001]), this pattern was not observed in the noninhibitor group (0.53 [0.0] before vs. 0.37 [0.0] with emicizumab, [*P* = 0.102] and 0.85 [0.0] before emicizumab vs. 0.28 [0.0] with emicizumab, [*P* = 0.320]). The percentage of persons achieving zero bleeds with emicizumab was significant in both groups, in the inhibitor group (63.0% vs. 22.2% before emicizumab, *P* < .001), and in those without inhibitors (58.7% vs. 28.3% before emicizumab, *P* < .001).

Nine patients with inhibitors were treated on demand before switching to emicizumab. We observed a substantial reduction in the overall ABR after the switch. The median ABR (IQR) decreased from 1.45 (0.00-2.68) to 0.00 (0.00-0.52).

We did not observe any new inhibitors in PUPs or MTPs during emicizumab treatment. Five children were given repeated doses of FVIII with intention to get tolerant to FVIII (up to 50 EDs given weekly or every other week), while checking for inhibitors after every 10 EDs. Only one patient was given 1 extra dose of FVIII to treat a bleed. Remaining 53.8% of the PUPs/MTPs were never given FVIII after switching to emicizumab, inhibitor status was assessed approximately once per year in them.

### 3.4 Treatment of bleeds, factor and BPA consumption per bleeding episode

In the total group of 73 persons treated with emicizumab, 177 bleeds were recorded before and 73 bleeds during emicizumab treatment. The total factor consumption (IU/kg) used for the management of breakthrough bleeds during the follow-up periods decreased significantly after emicizumab initiation: for SHLs from median (IQR) 113 (73-216) to 0.0 (0.0-7,0; *P* = .001), for EHLs from 68 (36-136) to 0.00 (0.0-68; *P* = .322). In persons with inhibitors, aPCC consumption prior to emicizumab was 1967 (1679-2291) IU/kg, it was never used to treat bleeding during emicizumab prophylaxis (*P* = .031), and rFVIIa consumption (μg/kg) during the follow-up periods decreased from median (IQR) 109 (0-934) to 95 (0-211), *P* = .339.

The factor and BPA consumption per bleeding episode is shown in Table 4.

**Table 4:** Factors and bypassing agents consumption to treat bleeds per patient.

	Before emicizumab *n* = 73	Emicizumab *n* = 73	*P*^a^
Length of FUP (months)			
*n*	73	73	
Mean ± SD	19 ± 14	30 ± 15	
Median (IQR)	15 (9-24)	30 (18-43)	
Sum	1393	2204	
SHL (IU/kg)			.001
*n*	28	28	
Mean ± SD	197 ± 208	53 ± 133	
Median (IQR)	113 (73-216)	0 (0-7)	
Sum	5528	1481	
EHL (IU/kg)			0.322
*n*	23	23	
Mean (SD)	101 ± 94	100 ± 187	
Median (IQR)	68 (36-136)	0 (0 – 68)	
Sum	2326	2294	
aPCC (IU/kg)			0.031
*n*	6	6	
Mean ± SD	2414 ± 2093	0 ± 0	
Median (IQR)	1967 (1679-2291)	0 (0-0)	
Sum	14,482	0	
rFVIIa (ug/kg)			.339
*n*	12	12	
Mean ± SD	3321 ± 7945	134 ± 157	
Median (IQR)	109 (0-934)	95 (0-211)	
Sum	39,848	1612	

aPCC, activated prothrombin complex concentrate; EHL, extended half-life FVIII; SHL, standard half-life FVIII; IQR, interquartile range; rFVIIa, recombinant activated factor VII.

a Wilcoxon signed rank exact test for comparison of consumption per kg.

### 3.5 Safety

Data on thromboembolic events or thrombotic microangiopathy were not specifically collected in the CNHP registry, but we actively asked every treating centers and no such events were observed/registered in the primary data source (patients’ files). The same also applies to antidrug antibodies.

All documented FVIII inhibitors developed during FVIII treatment prior to emicizumab initiation and no new or recurrent inhibitors occurred during the emicizumab treatment.

## 4 Discussion

Following the encouraging results of clinical trials and the approval of the drug, emicizumab quickly entered routine clinical use and, to some extent, fundamentally changed existing practice. Several reports have shown that clinical practice varies widely in terms of who is started on this nonfactor therapy and when [[14], [15], [16]]. In countries with access to emicizumab, it has undoubtedly become the new standard of care for persons with inhibitors, whereas in those without inhibitors, various practices have been observed [16]. Moreover, it has been hypothesized that this innovative treatment has the potential to provide freedom in different areas of life, resulting in a hemophilia-free mind [17].

The results of our analysis of data from the national registry confirmed the favorable safety and efficacy profile of emicizumab prophylaxis in adults and children. Consistent with the results of the HAVEN trials [[7], [8], [9]] and other real-world data reports [[18], [19], [20], [21]], a significant reduction in total ABR was observed with emicizumab in our cohort, in persons with and without inhibitors.

A recently published 3-year follow-up of nonselected persons of all ages with inhibitors from the UK National Haemophilia Database showed, in a within-person analysis, a similar significant reduction in total ABR (from mean 4.97 to 0.50 vs. 3.75 to 0.24 in our cohort) and in the rate of zero treated bleeds (from 45 to 88% vs. 22.2 to 63% in our cohort), compared to pre-emicizumab after the switch [22]. Regarding joint and spontaneous bleeding, a significant impact on bleeding reduction was observed in our cohort specifically in persons with inhibitors. Again, these results strongly support the dominant position of emicizumab in persons with inhibitors. All persons with inhibitors in the registry have been thus switched to emicizumab as a new gold standard of treatment.

It should be emphasized that data from the Czech National Haemophilia Programme showed that patients without inhibitors had already achieved very good bleeding control with previous factor prophylaxis, with a median total ABR of 1.28 and both AsBR and AjBR of 0.00. Therefore, the further reduction in bleeding rate after switch to emicizumab was small but still observable. A meta-analysis focusing on bleeding events in persons using prophylaxis showed a large existing variation in the measured mean parameters of bleeding control (ABR, AjBR, and zero bleeds) across the observed cohorts [23] and highlighted that there is still room for improvement in outcomes, but, very importantly, identified the need for a standardized system to capture the above mentioned parameters in order to be able to effectively compare different treatments. Based on our results, we can speculate that prophylaxis, with either modern FVIII factors or emicizumab, provides effective protection against bleeding in the majority of cases, and that in the near future, patient/family preferences, e.g. convenience of treatment or other factors, may become dominators in the choice of a particular agent for prophylaxis.

Of all the people without inhibitors in the registry, 24% were treated with emicizumab. The highest proportion was found in the youngest age group (80%), whereas emicizumab was prescribed to only 11% of adults. The median age of the noninhibitor group was 5 years (IQR 1-20). Though there was no strict national switching policy, emicizumab was preferably chosen for younger children in pediatric centers during the early phase of national roll-out. More than half of all persons (54.1%) were the youngest patients in the age group 0-4 years, of whom 17% were PUPs and 39.1% MTPs. As such, a substantial proportion of persons without inhibitors were only started on regular prophylaxis with emicizumab, which was preferred as a convenient approach mainly in children with difficult venous access or those with a high-risk family background for inhibitors development or those at risk for recurrent severe bleeds. This particular observation strongly supports the Journal of Thrombosis and Haemostasis in Clinic recommendation by Young et al. [24] for a clear preference of emicizumab in HA prophylaxis in various clinical scenarios, as opposed to the International Society on Thrombosis and Haemostasis recommendation for the HA management [25]. The same applies to the trend of starting emicizumab prophylaxis at an earlier age compared to previous practice. The effectiveness and ease of implementation of very early primary prophylaxis with emicizumab in daily practice still needs to be proven. However, the interim analysis of the HAVEN 7 study and real-world experience from Israel are very promising [15,26].

The young age may have had an impact on the lower AjBR observed in this group, with no previously present impaired joints. With a higher proportion of older persons treated with emicizumab, this parameter is likely to increase [27].

The total ABR and AsBR were higher in the younger age group before emicizumab initiation compared to persons 12+ years of age, which was not the case for AjBR. We assume that the bleeding events reported in younger children were predominantly minor trauma bleeds that did not require further treatment, but it appears that these events were also significantly reduced with the initiation of emicizumab. Again, this observation supports previously published data on bleeding patterns in younger children [27]. We found that the number of subjects with zero bleeds in the younger group almost tripled with emicizumab.

The dosing of emicizumab according to the recorded data was in line with the product label recommendation in the majority of centers. The most typical maintenance dosing schedule was every 14 days. In 5 cases, the dosing schedule was adopted with the aim of injecting a whole vial and adjusting the dosing interval. As previously reported [12,13], vial-centered dosing may become the preferred approach and some persons and/or their families may find such dosing an attractive option in order to further reduce the treatment burden.

In the group of persons with inhibitors, the registry data showed a significant reduction in the use of rFVIIa to treat breakthrough bleeding episodes, particularly in the age group 12 years and older. Prior to the switch to emicizumab, the majority of bleeding episodes required multiple injections of rFVIIa to achieve good bleeding control compared to the emicizumab period, when typically only 1 injection was given. A significant reduction in factor consumption was seen in the treatment of bleeding in persons without inhibitors. These outcomes may be reflected in the total cost of care for persons with HA. However, it is beyond the scope of this article to calculate the full economic impact of the changes associated with the introduction of nonfactor treatments into real clinical practice in the Czech Republic. This could be assessed in a longer follow-up period.

The limitations of this analysis are the retrospective study design and the reliance on investigator-assessed causes of bleeding. On the other hand, the inclusion of all pediatric and most adult centers in the registry and analysis of data from all consecutive persons with HA strengthens the claim of this analysis to describe the national trend in haemophilia A treatment in the Czech Republic.

## 5 Conclusion

Our study provides a consistent report nationally on the implementation of emicizumab in routine clinical practice. The retrospective analysis of data from the CNHP registry supports the potential of emicizumab to reduce bleeding tendency and provide the opportunity for a significant proportion of persons with and without FVIII inhibitors to achieve zero bleeds requiring no additional treatment, particularly in young persons. These data, combined with the demonstrated reduction in treatment burden with emicizumab regimens, demonstrate that emicizumab prophylaxis is a well-tolerated and effective treatment option for persons with haemophilia A of all ages in the Czech Republic.
